# Supplementary material for: Collaborative intelligence in AI: Evaluating the performance of a council of AIs on the USMLE
Source: PLOS Digit Health. 2025 Oct 9;4(10):e0000787. doi: 10.1371/journal.pdig.0000787 (PMC12510544; doi:10.1371/journal.pdig.0000787)
Supplement: S2 File — (ZIP) [file pdig.0000787.s002.zip › S2 Data Raw data files containing unprocessed question inputs and LLM outputs including transcripts of deliberations/STEP3_Question-27-1_Type-MC-NJ.docx]

**Facilitator** **[Round 1]:** A 46-year-old woman, gravida 1, para 1, comes to the office because of a 2-week history of black discharge from her right nipple. The patient had mammography and ultrasonography of the breasts 2 months ago for evaluation of increased glandularity, palpated in the upper outer quadrant of the right breast, noted at her most recent annual examination. The studies showed likely benign findings with recommended follow-up in 6 months. Medical history is otherwise unremarkable and she takes no medications. BMI is 30 kg/m2 . Vital signs are normal. Palpation of the right breast discloses glandularity in the upper outer quadrant but no other masses. There is scant, black discharge from the right nipple. Which of the following is the most appropriate next step in diagnosis? (A) Ductography (B) Excisional biopsy of glandular tissue (C) Repeat mammography (D) Repeat ultrasonography of the right breast (E) No further workup is indicated

**AI_C:** RESTATING THE QUESTION: The given clinical scenario revolves around a 46-year-old female patient who reports a 2-week history of black nipple discharge from her right breast. She recently underwent mammography and ultrasonography for evaluation of a palpable increased glandularity noted at her last annual exam. The results suggested possible benign abnormalities with a recommendation to reassess in 6 months. This patient's overall health context is otherwise normal, and findings from her physical examination are consistent with previous test results. The question seeks to determine the optimal subsequent diagnostic step from a list of possible interventions including ductography, excisional biopsy, repeat imaging studies, or advocating for no further investigation. PLANNING: While evaluating the mentioned patient scenario, there are multiple assumptions we need to consider. One assumption is that the occurrence of black discharge could indicate a pathological condition that was not detected in the previous imaging studies. An alternate assumption could be that this discharge is a harmless symptom, possibly related to a benign condition. The priority in clinical approach would be to rule out any potential malignancy given the new symptom of nipple discharge. CLINICAL REASONING: The black discharge from the nipple can be due to a variety of conditions, both benign and malignant. For instance, it could be due to a benign breast disease such as intraductal papilloma, duct ectasia, or could be suggestive of a more serious condition like carcinoma. However, the recent mammography and ultrasonography reports pointed towards likely benign findings. This diagnosis was corroborated by the absence of any additional breast masses upon palpation. Consequently, the foremost concern with the patient's black nipple discharge is to rule out an underlying malignant condition that might have been missed in the previous tests. CONCLUDING: To accurately determine the cause of the nipple discharge, visualization of the affected duct or ducts will likely provide the most useful information. Therefore, a diagnostic intervention that directly addresses this area seems the most prudent. RESPONSE: The most appropriate next step in diagnosis would be (A) Ductography. This procedure can provide direct imaging of the ductal system in the breast, helping to identify any underlying pathology that may be causing the black nipple discharge.

**AI_A:** 1) RESTATING THE QUESTION: The situation involves a 46-year-old female patient who has been experiencing black discharge from her right nipple for two weeks. The patient had previously undergone a mammography and ultrasonography for increased glandularity in her right breast's upper outer quadrant, with benign results. Despite these benign findings, she now presents a troubling symptom, and we need to determine the most appropriate next diagnostic step. 2) PLANNING: One assumption might be that we can simply wait for the six-month follow-up interval suggested by the previous imaging studies due to benign findings initially, however, the new symptom could suggest an overlooked pathology. Therefore, an alternative assumption is that additional diagnostic work-up is warranted immediately, as the black discharge from the nipple could indicate a more serious condition, such as ductal carcinoma in situ or intraductal papilloma, which wasn't picked up on initial imaging. 3) CLINICAL REASONING: The discrepancy between the benign findings on initial imaging and the appearance of black nipple discharge indicates that we may need to look more closely at the breast's structure, particularly the ducts. It may be that a microscopic pathology was missed on the original imaging. Additionally, the absence of other systemic symptoms might suggest a localized breast issue. Therefore, focusing on how the discharge is produced could be insightful. 4) CONCLUDING: Given the unexplained black discharge and the need for more specific insight into the breast duct's interior, a procedure that targets these structures should be considered, nonetheless, never forget the rationale and the potential risk associated with any intervention. Balancing the risk of missing a serious condition with the invasiveness of a specific examination is critical. 5) RESPONSE: Among the provided options, ductography (Option A) seems to be the most appropriate next step in the diagnostic process. In this case, It may aid in identifying any abnormalities within the breast ducts, such as a ductal carcinoma in situ or an intraductal papilloma, which could have been missed on previous mammography and ultrasonography.

**AI_B:** RESTATING THE QUESTION: This query is concerning a 46-year-old woman with a symptom of black discharge (pathologic nipple discharge) from the right nipple which has been present for two weeks. Despite past imaging studies indicating benign findings, the new symptom raises questions if the lesion is truly benign or if there has been a progression of a pre-existing benign condition to malignancy. PLANNING: I'm assuming that a new symptom, in this case the 'black discharge', indicates a possible change in the state of the breast lesion. However, other alternatives could be an incidental finding in an otherwise benign lesion, or a further benign condition not reflected in the conducted imaging tests. Evaluating these possibilities, it's crucial to identify if this new symptom could be indicative of a worsened or progressing condition or malignancy. CLINICAL REASONING: Pathologic nipple discharge (typically bloody, serous or black) can be a presenting symptom of breast cancer, but is more often associated with benign breast disease. Black discharge may be due to intraductal papilloma or ductal carcinoma in situ, although is most commonly associated with intraductal papillomata. Notably, mammograms may not detect an intraductal papilloma, explaining the previous benign findings. As these are located in the ducts near the areola, they can be only visualized by galactography (ductography), a procedure in which the ductal system is outlined by contrast material. Breast ultrasonography, which had also been performed previously, is less helpful as it would only show dilated ducts which is not enough for a definitive diagnosis. CONCLUDING: Given this context, my deduction is that ductography (option A) would be the most appropriate diagnostic step to take. This is based on the suspected presence of an intraductal papilloma given the presenting symptoms, and a ductography should provide the necessary imaging to confirm this. RESPONSE: The most appropriate next step in this patient's diagnosis would be to perform a ductography (Option A), as it can visualize the intraductal lesions causing the 'black discharge' symptom, which were not detected by previous imaging studies.

**AI_E:** (1) RESTATING THE QUESTION: The patient is a 46-year-old female with a recent symptom of black discharge from her right nipple. Upon examining her, very little black discharge can be seen from the right nipple, and there is an increased amount of glandular tissue in the upper outer quadrant of the right breast but no observable masses. Notably, her recent mammogram and ultrasound, done two months ago, revealed likely benign features, with a follow-up suggested in 6 months. Taking into account her age, symptom, physical examination, history of normal imaging studies, and the lack of any remarkable medical history, what should be the most suitable subsequent step for her diagnosis? (2) PLANNING: The clinical challenge in this question is to decide whether more imaging (i.e., a repeat mammogram or ultrasound), direct diagnostic procedures (i.e., ductography or excisional biopsy), or no further investigations are needed. In evaluating this, her age, symptom, physical exam, and recent imaging do not point towards an impending malignancy. Although she is at the age when most breast cancers occur, her symptom of black nipple discharge is often related to a benign breast condition, which is backed by her previously normal imaging studies. Additionally, her physical exam does not show any masses or suspicious findings other than glandularity. (3) CLINICAL REASONING: While an excisional biopsy could definitively identify whether the glandular tissue is benign or malignant, this invasive procedure should be reserved for suspicious findings or diagnostic uncertainty. Repeating the mammography or ultrasonography just two months after her previous likely benign studies introduces unneeded radiation exposure or may produce unnecessary anxiety due to possible false-positive findings. Additionally, without any further suspicious findings or history, we cannot justify daily imaging investigations based on a commonly benign symptom, especially in the absence of any other suspicious clinical findings. (4) CONCLUDING: As a result, the best next step would be a diagnostic procedure that could provide more information about the source and nature of the nipple discharge. Ductography or galactography is a minimally invasive imaging technique that involves the injection of contrast material into the lactiferous ducts followed by mammography. It is especially useful in evaluating nipple discharge cases as it can identify intraductal abnormalities, such as a single dilated duct, intraductal mass, or intraductal filling defect, which would guide further management. (5) RESPONSE: Thus, taking into consideration her clinical presentation, symptom, imaging findings, and the need for further evaluation without causing excessive discomfort or risk, option (A) Ductography would be the most appropriate next step in her diagnosis.

**AI_D:** (1) RESTATING THE QUESTION: To paraphrase the scenario, a 46-year-old woman with a previous pregnancy and a birth history (indicated by gravida 1, para 1) is concerned about two weeks of black discharge from her right nipple. Two months prior, she had a mammogram and breast ultrasound due to an increase in glandular tissue in the upper outer quadrant of her right breast. The findings were likely benign, and a follow-up was recommended in six months. She has no significant medical history, does not take any medications and has a BMI of 30 kg/m2. Physical examination of the breast reveals glandular tissue but no masses, with scant black discharge from the right nipple. The question is asking what the most appropriate next diagnostic step should be. (2) PLANNING: Factors to consider when approaching this problem include the patient's age, the characteristics of the nipple discharge (in this case, black), the presence of palpable glandularity without masses, and the benign findings on previous mammography and ultrasonography. Black nipple discharge might indicate a problem like duct papilloma or ductal carcinoma. Given that these clinical possibilities have distinctive management approaches, it's critical to clinically prioritise the assumption of the presence of underlying pathological process, even amid previous benign radiological findings. (3) CLINICAL REASONING: Nipple discharge in a non-lactating woman can be concerning for the patient and the physician. While the discharge is often benign in origin (for example, coming from an intraductal papilloma), it may also be a sign of malignancy (such as ductal carcinoma in situ). The fact that the mammography and ultrasonography from two months ago showed likely benign findings does not completely rule out malignancy, given the new symptom of nipple discharge. However, since the glandularity was already established before the onset of this discharge, a pathological process involving the ductal system of the breast might be a more likely cause, rather than just increased glandularity itself. (4) CONCLUDING: In this context, the aim is to characterize the nature of the nipple discharge, pinpoint its origin, and rule out malignancy. In order to accomplish this, a ductogram (or ductography), which is a targeted radiological technique that visualizes the breast ductal system, is particularly helpful and hence, will be the recommended next diagnostic step. (5) RESPONSE: Given that the patient's previous mammography and ultrasonography were unremarkable and given her new symptom of black nipple discharge, (A) Ductography is the most appropriate next step in diagnosis. This procedure will help visualize the breast ductal system in order to characterize the nature of the discharge and its source.

**Facilitator** The consensus among the AI responses is that the most appropriate next step in the diagnosis of the 46-year-old female patient, who has been experiencing black discharge from her right nipple for two weeks, is to perform a ductography (Option A). This conclusion is based on the fact that the patient's new symptom of black nipple discharge could indicate a pathological condition that was not detected in the previous imaging studies, such as ductal carcinoma in situ or intraductal papilloma. Despite the benign findings from her recent mammography and ultrasonography, the black discharge suggests that a more detailed examination of the breast's ductal system is warranted. Ductography is a minimally invasive procedure that can provide direct imaging of the ductal system in the breast, helping to identify any underlying pathology that may be causing the black nipple discharge. This approach balances the need to rule out a serious condition with the invasiveness of the examination, and it is considered the most prudent given the patient's clinical presentation.
